# Graves disease is associated with increased risk of clinical Alzheimer’s disease: evidence from the Medicare system

**DOI:** 10.1186/s40842-024-00170-z

**Published:** 2024-02-05

**Authors:** Arseniy Pavlovich Yashkin, Stanislav Kolpakov, Svetlana Ukraintseva, Anatoliy Yashin, Igor Akushevich

**Affiliations:** Biodemography of Aging Research Unit, Social Science Research Institute, Duke University, Room A115 Bay A, Erwin Mill Building, 2024 W. Main St., PO Box 90420, 27708 Durham, NC USA

**Keywords:** Graves disease, Alzheimer’s disease, Hyperthyroidism, Dementia, Medicare

## Abstract

**Background:**

Identification of modifiable risk factors for Alzheimer’s Disease (AD) onset is an important aspect of controlling the burden imposed by this disease on an increasing number of older U.S. adults. Graves disease (GD), the most common cause of hyperthyroidism in the U.S., has been hypothesized to be associated with increased AD risk, but there is no consensus. In this study, we explore the link between GD and risk of clinical AD.

**Methods:**

Cox and Fine-Grey models were applied to a retrospective propensity-score-matched cohort of 19,798 individuals with GD drawn from a nationally representative 5% sample of U.S. Medicare beneficiaries age 65 + over the 1991–2020 period.

**Results:**

Results showed that the presence of GD was associated with a higher risk of AD (Hazard Ratio [HR]:1.19; 95% Confidence Interval [CI]:1.13–1.26). Competing risk estimates were consistent with these findings (HR:1.14; CI:1.08–1.20) with the magnitude of associated risk varying across subgroups: Male (HR:1.25; CI:1.07–1.47), Female (HR:1.09; CI:1.02–1.16), White (HR:1.11; CI:1.03–1.19), and Black (HR:1.23; CI:1.02–1.49).

**Conclusions:**

Our results indicate a robust and consistent association between a diagnosis of GD and a subsequent diagnosis of AD in later stages of life. The precise biological pathways that could potentially connect these two conditions remain unclear as is the role of treatment in this relationship. Replications of these findings on datasets with both biomarkers and laboratory test results, especially in underrepresented groups is vital.

**Supplementary Information:**

The online version contains supplementary material available at 10.1186/s40842-024-00170-z.

## Background

Alzheimer’s disease (AD) is a debilitating neurodegenerative disorder, most prevalent in the elderly. Although AD is not part of the natural aging process, age is the most important non-genetic risk factor for the onset of this condition. AD places great strain on the U.S. healthcare system [1, 2] as well as on the health and financial well-being of family members and informal caregivers [1, 3–8]. At this time there is no clinically validated treatment available for AD other than palliative care; available pharmacological treatment options have uncertain efficacy [9–12] and are associated with significant monetary cost [13, 14]. Recent estimates place the prevalence of AD in U.S. older adults age 65+, at about 11,300 per 100,000 [15, 16]; although more prevalent in females [17, 18] the role of biological sex in AD risk is not straightforward [19]. In the future, AD is expected to exhibit rising incidence and post-onset mortality coupled with falling survival [20] leading to a projected increase in the prevalence of AD in the U.S [16, 21, 22].

Graves disease (GD) is an autoimmune disorder that causes overproduction of thyroid hormones which can accelerate the metabolism, leading to a weight loss, rapid heartbeat, and other symptoms; GD is the most common cause of hyperthyroidism in the U.S [23]. Current estimates of the prevalence of GD in the U.S. range between 20 and 50 per 100,000 [24] with women (approximately 40 per 100,000) being at higher risk than men (approximately 10 per 100,000) [23, 25]. Unlike AD, GD can be treated successfully. However, short of total thyroidectomy or radioactive iodine ablation [26], there is no permanent cure, with other treatments associated with high rates of recurrence and low rates of disease remission [23, 26, 27]. Although GD is not an aging-related disease per se, with incidence peaking between 30 and 50 years of age [23], some complications of GD are more common in the elderly [23, 28]. Increased risk of cognitive decline and the onset of AD/dementia have recently been associated with hyperthyroidism and GD [29–36] while results for hypothyroidism have been mixed [37–40]. If correct, then mitigation of the risk associated with GD, through timely identification and successful treatment, becomes an actionable policy target with clear benefits both directly to individuals with hyperthyroidism and indirectly to the public by reducing the magnitude of the burden associated with AD. In this study, we will explore the potential relationship between GD and the risk of clinical AD/dementia in later life through a comparison of propensity-score matched groups of U.S. older adults age 65+.

### Data and methods

Data came from a nationally representative 5% sample of Medicare beneficiaries provided by the U.S. Centers for Medicare and Medicaid Services (CMS). In the U.S., the Medicare social health insurance system pays for the healthcare of over 98% of the U.S. population age 65+. The dataset spanned the 1991–2020 time-period and provided individual-level information on the dates of birth and death (if applicable), race/ethnicity, sex, and the diagnoses made (using International Classification of Diseases 9th (ICD-9) and 10th (ICD-10) revision codes) during episodes of care paid for by Medicare Parts A (facility) or B (professional) over that period. We limited our analysis to individuals living within the U.S. and enrolled in either traditional fee-for-service Medicare or a Medicare Advantage plan whose claims are processed by the CMS. Most Medicare Advantage plans do not share claims data with the CMS and therefore information on their beneficiaries is not available for research.

For the calculation of trends in age-adjusted incidence and prevalence, we required the beneficiary to be age 65 + and aggregated all individuals older than 100 into the 100 + age group (this was done both to simplify the age-adjusting process and to better comply with data reporting restrictions set forth by the CMS). The resulting samples contained over 1,600,000 individuals for each study year. Although estimates for 1991–1993 are provided, these should be taken with care as many individuals in this period are still in the process of accumulating diagnoses after entry into the data. Incidence was calculated as the number of new cases of GD diagnosed before the end of a given year divided by the number of at-risk individuals present during that year. Prevalence was calculated as the number of living individuals diagnosed with GD on or before the end of a given year divided by the total number of living individuals. Age adjustment was done using the U.S. population for the year 2000 provided by the U.S. Census Bureau.

For the survival analysis portion of the study, we additionally required the presence of at least 3 years of look-back to ascertain the presence of baseline comorbidities, and at least one year of follow-up. The baseline age was set at the time of a verified GD diagnosis for the cases and three years after data entry for the controls. The look-back period was measured both from the individual age and from the calendar time perspective, making the minimum baseline age about 67.5 and the minimum baseline time January 01, 1994. Individuals with AD on or before the baseline date were excluded. The final sample pool for the survival analysis portion of the study consisted of 3,399,925 individuals.

The presence of co-morbid conditions from the Elixhauser co-morbidity index index [41], Graves Disease (ICD-9:242.00, 242.01; ICD-10:E05.00, E05.01), and Alzheimer’s Disease (ICD-9:331.0; ICD-10:G30), our primary outcome, were identified as follows: For AD, and GD we required at least two distinct claims no more than two years from each other. Date of onset was set at the earliest date of the two. This was motivated by the relative rarity of the conditions, and the need to mitigate the potential bias associated with erroneous diagnosis. Studies on GD relying on biologic data [42], have required the presence of two distinct serum thyroid-stimulating hormone concentration test values of < 0.3 mIU/L. Although our data does not have access to test results (therefore we do not observe if the < 0.3 mIU/L cut-off was reached), requiring a second episode of care with GD recorded as a diagnosis helps to approximate this process. Furthermore, the above criteria may itself miss individuals with rapid post-treatment normalization of thyroid-stimulating hormone concentration. To address this issue, we repeat our primary analysis for individuals with only a single Graved Disease claim on record. These would be excluded from the Graves disease group in primary analysis (Supplementary Appendix A). For the Elixhauser-based co-morbidity index a more standard requirement of 90 days between two distinct claims was used. We also included the following socio-demographic covariates: male sex; Black, Hispanic, and Other (including Asian, Native Americans/pacific islander) race/ethnicity, dual eligibility (as a proxy for poor economic status) and a yearly trend (to represent changes in technology and practice).

Of the 3,399,925 individuals eligible for the analysis, 19,852 were identified as GD cases. After comparing the summary statistics between these groups, we concluded that the GD group was too dissimilar from the healthy population for direct comparisons (Tables 1 and 2: *Unmatched Full Sample* column). Therefore, a Greedy Propensity Score Matching (PSM) algorithm [43, 44] was used to identify a comparable group of individuals from the healthy control pool. We used 1:1 matching without replacement [45] based on propensity scores generated by a logistic model designed to estimate the probability of having GD using the 31 Elixhauser co-morbidities and available demographic variables. In this way, we were able to identify 19,798 matched pairs for GD (Tables 1 and 2: *Matched Full Sample* column). To assess any differences in risk associated with race, ethnicity and/or sex we stratified the full sample into six race/ethnicity/sex-specific subgroups and re-ran the PSM algorithm. This resulted in the identification of 4,233 matched pairs for male, 15,534 for female, 16,488 for White, 1,958 for Black, 226 for Hispanic and 948 for individuals of Other races. All analysis in this study is repeated for each of these subgroups.

**Table 1:** Summary statistics

	Full sample	Matched sample
Baseline Age	71.70 (6.13)	74.81 (7.87)
Male	0.42 (0.49)	0.23 (0.42)
White	0.85 (0.36)	0.82 (0.39)
Black	0.08 (0.27)	0.10 (0.31)
Hispanic	0.02 (0.13)	0.02 (0.12)
Other	0.04 (0.21)	0.05 (0.23)
Ever Dual Eligible	0.19 (0.39)	0.22 (0.41)
Yearly Trend (2000 = 0)	-2.85 (8.81)	-5.70 (8.21)
Congestive heart failure	0.06 (0.24)	0.19 (0.39)
Cardiac arrhythmias	0.10 (0.30)	0.33 (0.47)
Valvular disease	0.04 (0.19)	0.14 (0.35)
Pulmonary circulation Disorders	0.01 (0.10)	0.04 (0.20)
Peripheral vascular disorders	0.06 (0.23)	0.16 (0.37)
Hypertension, uncomplicated	0.38 (0.49)	0.65 (0.48)
Hypertension, complicated	0.04 (0.19)	0.13 (0.34)
Paralysis	0.01 (0.09)	0.02 (0.13)
Other neurological disorders	0.02 (0.15)	0.06 (0.24)
Chronic pulmonary disease	0.11 (0.31)	0.26 (0.44)
Diabetes, uncomplicated	0.14 (0.35)	0.25 (0.43)
Diabetes, complicated	0.04 (0.20)	0.10 (0.30)
Hypothyroidism	0.08 (0.27)	0.42 (0.49)
Renal failure	0.02 (0.15)	0.07 (0.26)
Liver disease	0.01 (0.12)	0.05 (0.21)
Peptic ulcer disease excluding bleeding	0.01 (0.11)	0.03 (0.18)
AIDS/H1V	< 0.01 (0.02)	< 0.01 (0.02)
Lymphoma	< 0.01 (0.07)	0.01 (0.11)
Metastatic cancer	0.01 (0.09)	0.02 (0.14)
Solid tumor without metastasis	0.07 (0.26)	0.16 (0.36)
Rheumatoid arthritis/ collagen vascular diseases	0.03 (0.17)	0.09 (0.29)
Coagulopathy	0.01 (0.12)	0.05 (0.23)
Obesity	0.03 (0.16)	0.06 (0.23)
Weight loss	0.01 (0.12)	0.10 (0.30)
Fluid and electrolyte disorders	0.04 (0.21)	0.16 (0.37)
Blood loss anemia	0.01 (0.07)	0.02 (0.15)
Deficiency anemia	0.03 (0.16)	0.11 (0.31)
Alcohol abuse	0.01 (0.07)	0.01 (0.09)
Drug abuse	< 0.01 (0.04)	0.01 (0.07)
Psychoses	0.01 (0.09)	0.02 (0.14)
Depression	0.05 (0.22)	0.13 (0.34)
N	3,399,925	39,596
N Graves Disease	19,852	19,798
N Alzheimer’s Disease	320,508	3,846
N Dead	1,413,974	17,065

Numbers presented are sample means with standard deviations in parentheses

**Table 2:** Propensity score matching quality

	Unmached	Propensity score matched groups						
	Full sample	Full	Male	Female	White	Black	Hispanic	Other
Baseline Age	**40.39**	**-11.73**	-4.06	**-12.81**	**-12.10**	**-10.95**	-2.21	-8.98
Male	**-44.56**	-7.27	N.A.	N.A.	-7.45	-6.57	0.99	-5.20
White	-5.41	7.79	**14.19**	6.50	N.A.			
Black	8.25	-2.84	-4.69	-3.82				
Hispanic	-3.67	-5.42	-7.12	-6.94				
Other	2.26	-4.08	-8.42	-0.92				
Ever Dual Eligible	1.43	**-12.25**	**-23.02**	**-17.09**	**-13.25**	-6.88	< 0.01	-7.64
Yearly Trend (2000 = 0)	**-43.90**	**-17.27**	**-21.66**	**-20.49**	**-19.36**	**-15.31**	0.17	**-11.81**
Congestive heart failure	**36.45**	-6.84	**-10.62**	-7.02	-9.10	-3.87	-3.12	-7.81
Cardiac arrhythmias	**55.23**	-7.24	-5.08	-7.22	-7.96	-4.21	8.98	-6.39
Valvular disease	**35.79**	-5.02	-3.03	-4.25	-6.67	-1.75	-3.93	-6.49
Pulmonary circulation Disorders	**20.09**	-2.68	-3.09	-2.95	-3.06	0.90	4.78	-2.58
Peripheral vascular disorders	**31.67**	-6.75	**-10.37**	-8.02	-7.18	-3.62	-5.55	< 0.01
Hypertension, uncomplicated	**53.70**	-6.19	-4.67	-4.90	-7.49	1.37	-6.76	2.93
Hypertension, complicated	**32.61**	-3.67	-9.17	-5.23	-5.72	-4.18	-4.29	-7.09
Paralysis	8.16	-2.36	-3.39	-2.76	-4.27	0.00	8.60	0.00
Other neurological disorders	**17.38**	-2.76	-5.03	-4.51	-6.36	-1.38	3.94	-5.37
Chronic pulmonary disease	**35.08**	-8.59	**-13.86**	-7.85	**-11.83**	-3.52	0.95	-3.70
Diabetes, uncomplicated	**25.24**	-7.36	-9.51	-8.71	-8.42	-4.10	-9.19	-6.74
Diabetes, complicated	**21.13**	-4.02	-7.58	-5.61	-5.97	0.28	-1.17	-8.16
Hypothyroidism	**90.71**	6.23	-0.40	6.46	6.61	2.21	-2.71	3.50
Renal failure	**22.93**	-3.09	-7.93	-5.09	-3.71	-2.41	-8.95	-7.36
Liver disease	**16.80**	-3.87	-3.73	-3.50	-3.98	-2.30	-3.18	0.45
Peptic ulcer disease excluding bleeding	**13.68**	-1.83	-2.57	-2.00	-3.21	-1.89	-1.86	-1.98
AIDS/H1V	-0.05	-1.29	-3.26	-1.89	-2.36	6.40	< 0.01	4.59
Lymphoma	6.64	-1.61	-1.53	-2.97	-3.05	-2.91	< 0.01	5.59
Metastatic cancer	**10.51**	-1.92	-5.92	-0.91	-4.73	-5.47	5.44	< 0.01
Solid tumor without metastasis	**25.60**	-4.74	-3.73	-3.88	-5.82	-8.01	-1.57	1.61
Rheumatoid arthritis/ collagen vascular diseases	**25.60**	-2.91	-2.78	-2.43	-2.18	-4.09	9.34	1.27
Coagulopathy	**21.35**	-3.12	-2.94	-3.65	-3.27	-0.26	1.93	-6.30
Obesity	**14.35**	-3.60	-7.12	-4.13	-4.85	2.11	-1.50	-1.13
Weight loss	**37.93**	-2.12	-3.19	-4.27	-2.32	-3.24	< 0.01	-3.42
Fluid and electrolyte disorders	**38.26**	-4.50	-7.95	-6.81	-7.36	-4.33	-5.46	-1.85
Blood loss anemia	**15.78**	-0.13	-0.89	-0.90	-2.00	-1.91	**11.00**	< 0.01
Deficiency anemia	**31.46**	-2.89	-3.41	-5.87	-3.54	-2.77	5.73	-3.48
Alcohol abuse	1.83	-3.95	-4.95	-3.85	-3.97	-1.68	< 0.01	1.53
Drug abuse	5.42	-1.59	-1.85	-1.85	-2.50	-2.62	-5.44	< 0.01
Psychoses	7.48	-3.63	-7.35	-3.54	-4.12	-1.36	< 0.01	-1.89
Depression	**26.35**	-5.29	-7.49	-7.14	-7.67	-3.69	1.29	< 0.01
N	3,399,925	39,596	8,466	31,068	32,976	3,916	452	1,896
N Graves Disease	19,852	19,798	4,233	15,534	16,488	1,958	226	948
N Alzheimer’s Disease	320,508	3,846	646	3,306	3,238	437	52	174

Numbers presented are standardized differences; Standardized differences with an absolute value greater than 10 are in bold

The standardized difference [46] was used to assess the inter-group differences before and after PSM. The standardized difference is not affected by differences in sample size and has the benefit of being relatable to two other measures of association, the Pearson correlation coefficient for continuous and the phi coefficient for dichotomous variables [47, 48]. We used the criterion of $\left|{\varDelta }_{s}\right|\le 10$% to reduce the inter-group differences to a level sufficient for further analysis. Using this criterion, we judged that the PSM algorithm successfully reduced the inter-group differences to a level sufficient for further analysis (Table 2). There were some exceptions. The baseline ages for the GD group occurred 11.73 (Full sample), 12.81 (Female sample), 12.10 (White sample) and 10.96 (Black sample) percentage points (pp) earlier on average than in their PSMed counterparts. None of these differences were greater than 1 year in real terms and this difference in age was explicitly addressed by the way we utilized age in our survival analysis models. The baseline dates for the GD group also occurred between 6.88pp (Black sample) to 23.02pp (Male sample) earlier than those of their PSMed counterparts. However, none of these were greater than 2 years, and most under one year, in real terms. Individuals with GD were 6.88pp (Black sample) to 23.02pp (Male sample) percentage points less likely to be dual eligible than their PSMed counterparts. There were also some minor differences in the rates of chronic pulmonary diseases in the White (-11.83pp) and Male (-13.86pp) samples; peripheral vascular disorders in the Male (-10.3) sample; and blood loss/anemia in the Hispanic (+ 11.00) sample.

Survival analysis was done using two methods: the Cox proportional hazards model and the Fine-Gray competing risk model [49] with death as the competing risk. In both cases, age, the most important non-genetic risk factor for AD, was included non-parametrically as a time-scale variable. Thus, the partial likelihood is maximized for individuals with the same value of the time scale variable. Therefore, the effects of age in the model are accounted for non-parametrically and, in a certain sense, exactly. The only covariate explicitly included in the model was membership in the GD group. The PSM matching ensures that the GD and non-GD groups were nearly identical in terms of all other covariates at baseline. All analysis was done using SAS 9.4 software (Cary, NC: SAS Institute Inc.) after obtaining permission from the Duke University IRB.

## Results

**Fig. 1:**
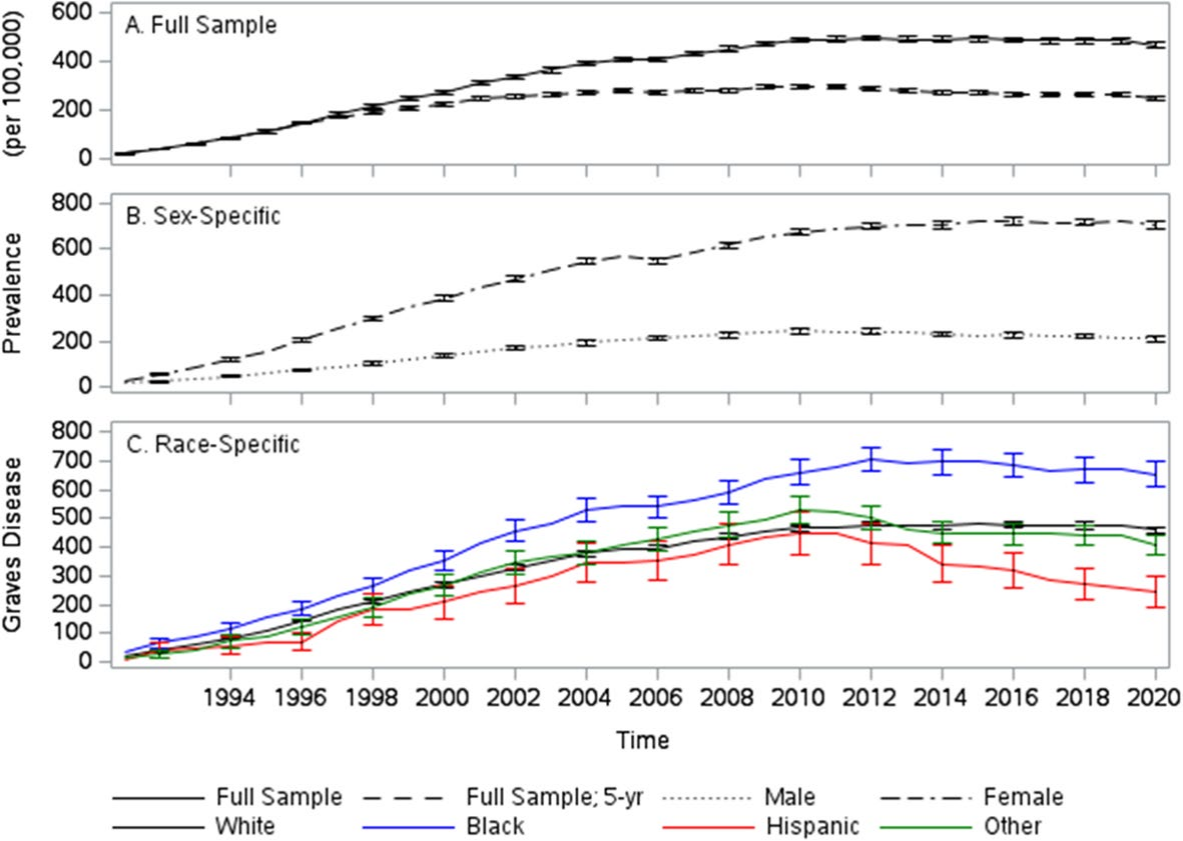
Trends in graves disease prevalence. Trend in the prevalence of individuals ever to be diagnosed with Graves Disease (per 100,000). Full sample, Graves Disease an absorbing state (**A **black solid line); Full sample, Graves Disease in remission after 5 years (**A **black dashed line); Males **B **black dotted line), Females (**B** black dot-dash line), White (**C** black solid line), Black (**C **blue solid line), Hispanic (**C** red solid line), other (**C** green solid line)

The total, 65 + age-adjusted prevalence of GD grew over the study period reaching a maximum of 495 per 100,000 in 2012 (Fig. 1A). Note that the nature of GD (e.g., need for long-term treatment; high recurrence and low remission rates), combined with the low accuracy of identifying remission from administrative health data led to the decision to treat GD as a permanent condition. Therefore, the prevalence levels are likely to be overestimated. Making a counterfactual assumption, that all instances of GD are cured over 5 years, the initial estimates fall sharply (Fig. 1A). As expected, (Fig. 1B) the prevalence of GD is significantly higher in females (maximum of 726 per 100,000 in 2019) than in males (maximum of 244 per 100,000 in 2010). Black individuals (Fig. 1C) have the highest prevalence of GD among all races/ethnicities (maximum of 705 per 100,000 in 2012) and this difference is statistically significant from all other groups from 2000 onwards. In contrast, Hispanic individuals have the lowest prevalence of GD (maximum of 450 per 100,000 in 2011). However, these differences are not statistically different from other non-Black races/ethnicities until 2014.

**Fig. 2:**
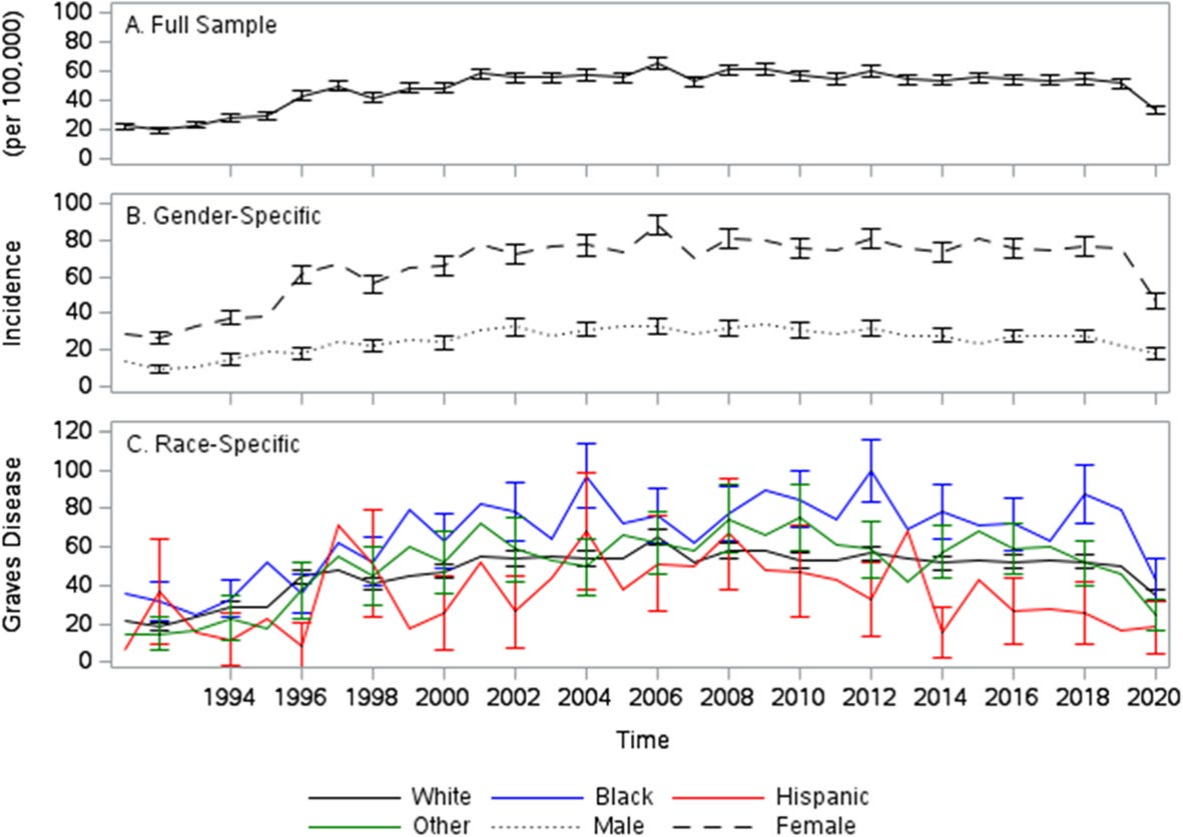
Trends in graves disease incidence. Trend in the incidence of Graves Disease (per 100,000).  Full sample (**A **black solid line) Males (**B **black dotted line), Females (**B **black dash line), White (**C **black solid line), Black (**C **blue solid line), Hispanic (**C **red solid line), other (**C **green solid line)

The total 65 + age adjusted incidence of GD, although subject to some fluctuations, is fairly constant with a maximum of 65 per 100,000 in 2006 (Fig. 2A). Like with prevalence, GD incidence (Fig. 2A) in females (maximum of 88 per 100,000 in 2006) is significantly higher than that of males (maximum of 33 in 100,000 in 2006). However, unlike prevalence, no strong race/ethnicity-related patterns in incidence can be observed (Fig. 2C). Black individuals have the highest incidence rates (maximum of 100 per 100,000 in 2012) and Hispanic the lowest. However, the race/ethnicity-specific confidence intervals overlap. Only in 2012, can we say with any statistical confidence, that Black incidence rates of GD are higher than those of the Hispanic group.

Survival analysis results are presented in Table 3. The Cox model shows that in a PSM sample, the presence of Graves disease is associated with higher risk of AD in the full sample (Hazard Ratio [HR]:1.19; 95% Confidence Interval [CI]:1.13–1.26), as well as the Male (HR:1.23; CI:1.03–1.47), Female (HR:1.17; CI:1.08–1.25), and White (HR:1.14; CI:1.06–1.23) groups. The results of the competing risk model, are lower on average, and highly consistent with those of the traditional Cox model. The association between GD and AD risk in Black individuals (HR:1.23; CI:1.02–1.49) becomes significant once the competing risk of death is accounted for. The hazard ratios obtained in sensitivity analysis described in Supplementary Appendix A are consistent with the primary results and the effect direction and confidence intervals of the subgroups for which a statistically significant effect could not be identified was consistent with significant findings. No race/ethnicity/sex-related disparities in the effect of GD on AD could be observed as the CI for all study subgroups overlap.

**Table 3:** Survival analysis results

	Full sample	Male	Female	White	Black	Hispanic	Other	Sensitivity
Cox	1.19^b^	1.23^a^	1.17^b^	1.14^b^	1.19	1.55	1.18	1.21^b^
	[1.13–1.26]	[1.03–1.47]	[1.08–1.25]	[1.06–1.23]	[0.96–1.46]	[0.78–3.08]	[0.84–1.64]	[1.13–1.30]
Competing Risk	1.14^b^	1.25^b^	1.09^a^	1.11^b^	1.23^a^	0.89	1.12	1.14^b^
	[1.08–1.20]	[1.07–1.47]	[1.02–1.16]	[1.03–1.19]	[1.02–1.49]	[0.52–1.53]	[0.83–1.51]	[1.08–1.20]
N Matched Pairs	19,798	4,233	15,534	16,488	1,958	226	948	23,706

^a^significant at the alpha = 0.05 level

^b^significant at the alpha = 0.01 level

## Discussion

In this paper we found that the presence of GD is associated with a significantly higher risk of clinical AD. Hyperthyroidism is a medical condition where thyroid-stimulating hormone (TSH) levels are low or even undetectable with normal free thyroxine and total or free triiodothyronine levels. It can be caused by increased endogenous production of thyroid hormone, as well as because of administration of thyroid hormone to treat malignant thyroid disease, or by excessive replacement therapy [50]. Association of TSH with dementia [51], suggests that prolonged exposure to low TSH levels could be detrimental to brain function. Individuals with goiter, hypothyroidism, thyroiditis, or hyperthyroidism faced an increased risk of AD, particularly in younger age groups, females, and those with lower comorbidity scores [52]. This, in turn, suggests that the level of estrogen is associated with thyroid function which was confirmed by studies, including ours, that showed that thyroid disorders are more prevalent in women than in men [53].

Another potential mechanism connecting GD (and related hyperthyroidism) to AD may involve shared etiological factors between the two diseases, such as viral infections, compromised/auto immunity, and neuroinflammation, which may themselves contribute to the development of both GD and AD [35, 54–58]. Neuroinflammation of the microglia, the brain’s resident macrophages [59], has been suggested to play a central role in the pathophysiological processes in both GD and AD [60–62] and hyperthyroidism was shown to aggravate cognitive deficits in AD mice and induce Aβ deposition and neuronal loss by inducing neuroinflammation [60].

Findings showing that both low and high thyrotropin (a pituitary hormone that stimulates the production of thyroid hormones) could be associated with increased risk of AD [32], suggest the possibility of a U-shaped relationship. This mechanism is supported by significantly higher prevalence rates of both hypothyroidism and hyperthyroidism in participants with AD found among a sample of the Korean National Health Insurance beneficiaries [52], and the lack of association between hyperthyroidism and dementia found in some studies [63, 64].

The above suggests that the role of metabolism could also be contextual [65, 66]. For example, slowdowns in metabolism might promote dementia through declining rates of information processing or by impairing the resilience of the body to adverse health events, such as infections, through delayed immune responses, slower healing, and longer recovery time [66]. On the other hand, it may also slow some aging-related changes in the body and be beneficial in the long term by, for example, reducing the rate of deterioration of the cortex and white matter in the brain [65, 66].

This study has several strengths. First, it was conducted after equalizing the GD and non-GD groups across a wide range of demographic and health-related conditions. This is vital as the GD group was shown to be statistically different from the non-GD population across many health-related conditions, including AD risk-related diseases. Second, it is based on data nationally representative of the 65 + population with follow-up periods of 27 + years. This provides the study with a relatively large group of individuals with a clinical diagnosis of GD even in smaller population strata. However, the study is based on administrative claims data designed for billing purposes, not research. Therefore, valuable information, such as the results of laboratory analyses, is not available. However, the disease ascertainment algorithm used is designed to reduce the impact of tentative and mistaken diagnoses (essentially we are assuming that if the laboratory results were not consistent with a diagnosis of GD, then it would not be listed as a diagnosis on the second visit) and our estimates of the extra risk of AD onset associated with GD are highly consistent with the estimates of at least one study based on biological measurements [42].

Similarly, AD, in the context of our data, represents a clinical diagnosis of *possible/probable Alzheimer’s Disease dementia*, and may not reflect the exact etiology of the individual’s actual condition. AD is often mistaken for other conditions and, often co-exists with other types of dementia making its diagnosis before autopsy difficult. Even though the study is nationally representative, and the sample size reflects the true situation as capturable by this dataset, additional studies with a focus on oversampling minority groups are warranted. Finally, we were not able to differentiate between the effects of treated/controlled GD and situations where disease management is proving a challenge or of any additional risk associated with alternative types of GD treatment. This is an important avenue of future research as studies have shown that for some chronic health conditions related to AD risk, aggressive management of the risk-related disease acts to reduce the associated AD risk as well [67].

## Conclusion

Although the exact biological mechanisms potentially linking the two conditions are unclear and focused studies of race/ethnicity-specific subgroups as well as the replication of these findings on datasets with available biomarkers and laboratory test results are needed, our findings support the hypothesis that there may be a strong relationship between a diagnosis of GD and a diagnosis of AD in later life.

### Supplementary Information

**Additional file 1:** **Supplementary Appendix A. Supplementary Table 1.  **Summary Statistics for Unconfirmed Graves Disease. **Supplementary Table 2.  **Propensity Score Matching Quality and Group Comparison for Unconfirmed Graves. **Supplementary Table 3. **Logistic Group Membership Models. **Supplementary Figure S1. **Trends in Unconfirmed graves disease prevalence.  **Supplementary Figure S2.** Trends in Unconfirmed graves disease incidence.

## Data Availability

This article is based on restricted data, not publicly available, but can be obtained from the Centers for Medicare and Medicaid Services.

## Abbreviations

AD: Alzheimer’s Disease
CI: 95% Confidence interval
CMS: Centers for Medicare and Medicaid Services
GD: Graves Disease
HR: Hazard Ratio
ICD: International Classification of Disease
PSM: Propensity Score Matching

## References

[1] Alzheimer’s A (2015). 2015 Alzheimer’s disease facts and figures. Alzheimers Dement.

[2] Hurd MD, Martorell P, Delavande A, Mullen KJ, Langa KM (2013). Monetary costs of dementia in the United States. N Engl J Med.

[3] Vitaliano PP, Zhang J, Scanlan JM (2003). Is caregiving hazardous to one’s physical health? A meta-analysis. Psychol Bull.

[4] Schulz R, O’Brien AT, Bookwala J, Fleissner K (1995). Psychiatric and physical morbidity effects of dementia caregiving: prevalence, correlates, and causes. Gerontologist.

[5] Mausbach BT, Chattillion EA, Roepke SK, Patterson TL, Grant I (2013). A comparison of psychosocial outcomes in elderly Alzheimer caregivers and noncaregivers. Am J Geriatr Psychiatry.

[6] Seeher K, Low LF, Reppermund S, Brodaty H (2013). Predictors and outcomes for caregivers of people with mild cognitive impairment: a systematic literature review. Alzheimer’s Dement J Alzheimer’s Assoc.

[7] Schulz R, Sherwood PR (2008). Physical and mental health effects of family caregiving. Am J Nurs.

[8] Kim Y, Schulz R (2008). Family caregivers’ strains: comparative analysis of cancer caregiving with dementia, diabetes, and frail elderly caregiving. J Aging Health.

[9] Walsh S, Merrick R, Milne R, Brayne C. Aducanumab for Alzheimer’s disease? British Medical Journal Publishing Group; 2021. 10.1136/bmj.n1682 PMC8258645 34226181

[10] Cummings J, Aisen P, Lemere C, Atri A, Sabbagh M, Salloway S (2021). Aducanumab produced a clinically meaningful benefit in association with amyloid lowering. Alzheimers Res Ther.

[11] Schneider L (2020). A resurrection of aducanumab for Alzheimer’s disease. Lancet Neurol.

[12] Alexander GC, Emerson S, Kesselheim AS (2021). Evaluation of aducanumab for Alzheimer disease: scientific evidence and regulatory review involving efficacy, safety, and futility. JAMA.

[13] Crosson FJ, Covinsky K, Redberg RF (2021). Medicare and the shocking US food and drug administration approval of aducanumab: crisis or opportunity?. JAMA Intern Med.

[14] Mafi JN, Leng M, Arbanas JC, Tseng C-H, Damberg CL, Sarkisian C, et al. editors. Estimated annual spending on Aducanumab in the US Medicare Program. JAMA Health Forum; 2022: American Medical Association. 10.1001/jamahealthforum.2021.4495 PMC8903103 35977233

[15] As A (2016). 2016 Alzheimer’s disease facts and figures. Alzheimers Dement.

[16] Rajan KB, Weuve J, Barnes LL, McAninch EA, Wilson RS, Evans DA (2021). Population estimate of people with clinical Alzheimer’s disease and mild cognitive impairment in the United States (2020–2060). Alzheimers Dement.

[17] Zhao L (2020). Alzheimer’s disease facts and figures. Alzheimers Dement.

[18] Ferretti MT, Iulita MF, Cavedo E, Chiesa PA, Schumacher Dimech A, Santuccione Chadha A (2018). Sex differences in Alzheimer disease—the gateway to precision medicine. Nat Rev Neurol.

[19] Akushevich I, Kravchenko J, Yashkin A, Doraiswamy PM, Hill CV, As D (2023). Expanding the scope of health disparities research in Alzheimer’s disease and related dementias: recommendations from the leveraging existing data and analytic methods for health disparities research related to aging and Alzheimer’s disease and related dementias workshop series. Alzheimers Dement.

[20] Akushevich I, Yashkin A, Kovtun M, Kravchenko J, Arbeev K, Yashin AI (2023). Forecasting prevalence and mortality of Alzheimer’s disease using the partitioning models. Exp Gerontol.

[21] Taylor DH, Sloan FA, Murali DP (2004). Marked increase in Alzheimer’s disease identified in medicare claims records between 1991 and 1999. J Gerontol A Biol Sci Med Sci.

[22] Babulal GM, Quiroz YT, Albensi BC, Arenaza-Urquijo E, Astell AJ, Babiloni C (2019). Perspectives on ethnic and racial disparities in Alzheimer’s disease and related dementias: update and areas of immediate need. Alzheimers Dement.

[23] Smith TJ, Hegedüs L (2016). Graves’ disease. N Engl J Med.

[24] Zimmermann MB, Boelaert K (2015). Iodine deficiency and thyroid disorders. Lancet Diabetes Endocrinol.

[25] Hussain YS, Hookham JC, Allahabadia A, Balasubramanian SP (2017). Epidemiology, management and outcomes of Graves’ disease—real life data. Endocrine.

[26] Wiersinga WM (2019). Graves’ disease: can it be cured?. Endocrinol Metab.

[27] Brito JP, Payne S, Singh Ospina N, Rodriguez-Gutierrez R, Maraka S, Sangaralingham LR (2020). Patterns of use, efficacy, and safety of treatment options for patients with Graves’ disease: a nationwide population-based study. Thyroid.

[28] Bahn RS (2010). Graves’ ophthalmopathy. N Engl J Med.

[29] Rieben C, Segna D, da Costa BR, Collet T-H, Chaker L, Aubert CE (2016). Subclinical thyroid dysfunction and the risk of cognitive decline: a meta-analysis of prospective cohort studies. J Clin Endocrinol Metabolism.

[30] Chaker L, Wolters FJ, Bos D, Korevaar TI, Hofman A, van der Lugt A (2016). Thyroid function and the risk of dementia: the Rotterdam Study. Neurology.

[31] Aubert CE, Bauer DC, da Costa BR, Feller M, Rieben C, Simonsick EM (2017). The association between subclinical thyroid dysfunction and dementia: the Health, Aging and Body Composition (Health ABC) Study. Clin Endocrinol.

[32] Tan ZS, Beiser A, Vasan RS, Au R, Auerbach S, Kiel DP (2008). Thyroid function and the risk of Alzheimer disease: the Framingham Study. Arch Intern Med.

[33] Bavarsad K, Hosseini M, Hadjzadeh MA, Sahebkar A (2019). The effects of thyroid hormones on memory impairment and Alzheimer’s disease. J Cell Physiol.

[34] Folkestad L, Brandt F, Lillevang-Johansen M, Brix TH, Hegedüs L (2020). Graves’ Disease and toxic nodular goiter, aggravated by duration of hyperthyroidism, are associated with Alzheimer’s and vascular dementia: a registry-based long-term follow-up of two large cohorts. Thyroid.

[35] Li X, Sundquist J, Zöller B, Sundquist K (2018). Dementia and Alzheimer’s disease risks in patients with autoimmune disorders. Geriatr Gerontol Int.

[36] Rieben C, Segna D, Da Costa BR, Collet T-H, Chaker L, Aubert CE, et al. Thyroid dysfunction and the risk of dementia and cognitive decline: systematic review, meta-analysis and clinical implications. 2016. 10.1210/jc.2016-2129 PMC6287525 27689250

[37] Thvilum M, Brandt F, Lillevang-Johansen M, Folkestad L, Brix TH, Hegedüs L (2021). Increased risk of dementia in hypothyroidism: a Danish nationwide register‐based study. Clin Endocrinol.

[38] George KM, Lutsey PL, Selvin E, Palta P, Windham BG, Folsom AR (2019). Association between thyroid dysfunction and incident dementia in the atherosclerosis risk in communities neurocognitive study. J Endocrinol Metab.

[39] Ma L-Y, Zhao B, Ou Y-N, Zhang D-D, Li Q-Y, Tan L (2023). Association of thyroid disease with risks of dementia and cognitive impairment: a meta-analysis and systematic review. Front Aging Neurosci.

[40] Han S, Jeong S, Choi S, Park SJ, Kim KH, Lee G, et al. Association of thyroid hormone medication adherence with risk of dementia. J Clin Endocrinol Metab. 2023:dgad447. 10.1210/clinem/dgad447 37515589

[41] Southern DA, Quan H, Ghali WA (2004). Comparison of the Elixhauser and Charlson/Deyo methods of comorbidity measurement in administrative data. Med Care.

[42] Papaleontiou M (2020). Higher risks of dementia in untreated and undertreated individuals with hyperthyroidism. Clin Thyroidol.

[43] Parsons L, editor. Reducing bias in a propensity score matched-pair sample using greedy matching techniques. Proceedings of the twenty-sixth Annual SAS users group international conference 2001. SAS Institute Inc.; 2001.

[44] Austin PC (2014). A comparison of 12 algorithms for matching on the propensity score. Stat Med.

[45] Austin PC, Small DS (2014). The use of bootstrapping when using propensity-score matching without replacement: a simulation study. Stat Med.

[46] Tritchler D (1995). Interpreting the standardized difference. Biometrics.

[47] Austin PC (2009). Using the standardized difference to compare the prevalence of a binary variable between two groups in observational research. Commun Stat Simul Comput.

[48] Austin PC (2009). Balance diagnostics for comparing the distribution of baseline covariates between treatment groups in propensity-score matched samples. Stat Med.

[49] Fine JP, Gray RJ (1999). A proportional hazards model for the subdistribution of a competing risk. J Am Stat Assoc.

[50] Donangelo I, Suh SY (2017). Subclinical hyperthyroidism: when to consider treatment. Am Fam Phys.

[51] Cooper DS, Samuels MH (2020). Hyperthyroidism and dementia. Thyroid.

[52] Kim JH, Lee HS, Kim YH, Kwon MJ, Kim J-H, Min CY (2022). The association between thyroid diseases and Alzheimer’s disease in a national health screening cohort in Korea. Front Endocrinol.

[53] Barth C, Crestol A, de Lange AMG, Galea LA (2023). Sex steroids and the female brain across the lifespan: insights into risk of depression and Alzheimer’s disease. Lancet Diabetes Endocrinol.

[54] Fulop T, Tripathi S, Rodrigues S, Desroches M, Bunt T, Eiser A (2021). Targeting impaired antimicrobial immunity in the brain for the treatment of Alzheimer’s disease. Neuropsychiatr Dis Treat.

[55] Yashin AI, Fang F, Kovtun M, Wu D, Duan M, Arbeev K (2018). Hidden heterogeneity in Alzheimer’s disease: insights from genetic association studies and other analyses. Exp Gerontol.

[56] Lim B, Prassas I, Diamandis EP (2021). From the amyloid hypothesis to the autoimmune hypothesis of Alzheimer’s disease. Diagnosis.

[57] Morshed SA, Latif R, Davies TF (2012). Delineating the autoimmune mechanisms in Graves’ disease. Immunol Res.

[58] Ukraintseva SV, Yashkin AP, Akushevich I, Arbeev KG, Duan H, Gorbunova GA, et al. Associations of infections and vaccines with Alzheimer’s disease point to a major role of compromised immunity rather than specific pathogen in AD. medRxiv. 2023:2023.12. 04.23299092. 10.1016/j.exger.2024.112411 PMC11060001 38548241

[59] Badimon A, Strasburger HJ, Ayata P, Chen X, Nair A, Ikegami A (2020). Negative feedback control of neuronal activity by microglia. Nature.

[60] Lou K, Liu S, Zhang F, Sun W, Su X, Bi W (2023). The effect of hyperthyroidism on cognitive function, neuroinflammation, and necroptosis in APP/PS1 mice. J Translational Med.

[61] Noda M (2018). Thyroid hormone in the CNS: contribution of neuron–glia interaction. Vitam Horm.

[62] Wang C, Zong S, Cui X, Wang X, Wu S, Wang L (2023). The effects of microglia-associated neuroinflammation on Alzheimer’s disease. Front Immunol.

[63] Van Vliet NA, Van Heemst D, Almeida OP, Åsvold BO, Aubert CE, Bae JB (2021). Association of thyroid dysfunction with cognitive function: an individual participant data analysis. JAMA Intern Med.

[64] Dolatshahi M, Salehipour A, Saghazadeh A, Sanjeari Moghaddam H, Aghamollaii V, Fotouhi A (2023). Thyroid hormone levels in Alzheimer disease: a systematic review and meta-analysis. Endocrine.

[65] Ukraintseva S, Yashin A, Arbeev K, Kulminski A, Akushevich I, Wu D (2016). Puzzling role of genetic risk factors in human longevity:risk alleles as pro-longevity variants. Biogerontology.

[66] Ukraintseva S, Arbeev K, Duan M, Akushevich I, Kulminski A, Stallard E (2021). Decline in biological resilience as key manifestation of aging: potential mechanisms and role in health and longevity. Mech Ageing Dev.

[67] Yashkin AP, Akushevich I, Ukraintseva S, Yashin A (2018). The effect of adherence to screening guidelines on the risk of Alzheimer’s disease in elderly individuals newly diagnosed with type 2 diabetes mellitus. Gerontol Geriatric Med.

