# Supplementary material for: Graves disease is associated with increased risk of clinical Alzheimer’s disease: evidence from the Medicare system
Source: Clin Diabetes Endocrinol. 2024 Feb 5;10:11. doi: 10.1186/s40842-024-00170-z (PMC10840251; doi:10.1186/s40842-024-00170-z)

**Supplementary Appendix A**

In this Appendix, we replicate the results of the main paper for individuals who received a single claim with a diagnosis of Graves Disease (GD), but for whom no further confirmation of GD status was available. This group potentially spans individuals with an erroneous claim for GD, individuals with a single abnormal TSH value; or alternatively individuals with rapid post-treatment normalization of thyroid-stimulating hormone concentration. Supplementary Table 1 and 2 present a comparison of the unconfirmed Graves group used for this sensitivity study and the sample used in the primary paper as well as the information presented in Tables 1 & 2 of the main paper. Supplementary Table 3, presents the results of the propensity score matching models for membership in the Confirmed and Unconfirmed Graves Group. In general, the unconfirmed Graves group was healthier, on average, than the confirmed Graves group, though the differences in terms of the individual conditions of the Elixhauser Index, the differences were slight.

| **Supplementary Table 1. Summary Statistics for Unconfirmed Graves Disease**        **Graves Disease Confirmation Status** | | | | |
| --- | --- | --- | --- | --- |
|  | **Full Sample** | **Matched Sample** | **Confirmed Graves** | **Unconfirmed Graves** |
|  |  |  |  |  |
| Baseline Age | 71.71 (6.14) | 76.20 (8.20) | 74.38 (7.22) | 75.99 (7.32) |
| Male | 0.42 (0.49) | 0.25 (0.43) | 0.22 (0.41) | 0.25 (0.43) |
| White | 0.85 (0.36) | 0.82 (0.38) | 0.83 (0.37) | 0.84 (0.37) |
| Black | 0.08 (0.27) | 0.10 (0.30) | 0.10 (0.30) | 0.09 (0.29) |
| Hispanic | 0.02 (0.13) | 0.02 (0.13) | 0.01 (0.11) | 0.02 (0.12) |
| Other | 0.04 (0.21) | 0.05 (0.22) | 0.05 (0.22) | 0.05 (0.22) |
| Ever Dual Eligible | 0.19 (0.39) | 0.24 (0.43) | 0.19 (0.39) | 0.21 (0.41) |
| Yearly Trend (2000=0) | -2.85 (8.80) | -4.96 (7.97) | -6.43 (7.54) | -5.58 (7.16) |
| Congestive heart failure | 0.06 (0.24) | 0.21 (0.41) | 0.17 (0.38) | 0.20 (0.40) |
| Cardiac arrhythmias | 0.10 (0.30) | 0.32 (0.47) | 0.31 (0.46) | 0.32 (0.47) |
| Valvular disease | 0.04 (0.19) | 0.17 (0.37) | 0.14 (0.34) | 0.17 (0.37) |
| Pulmonary circulation Disorders | 0.01 (0.10) | 0.04 (0.20) | 0.04 (0.20) | 0.04 (0.20) |
| Peripheral vascular disorders | 0.06 (0.23) | 0.19 (0.40) | 0.15 (0.36) | 0.19 (0.39) |
| Hypertension, uncomplicated | 0.38 (0.49) | 0.69 (0.46) | 0.64 (0.48) | 0.70 (0.46) |
| Hypertension, complicated | 0.04 (0.19) | 0.16 (0.37) | 0.13 (0.33) | 0.16 (0.36) |
| Paralysis | 0.01 (0.09) | 0.02 (0.15) | 0.02 (0.13) | 0.02 (0.14) |
| Other neurological disorders | 0.02 (0.15) | 0.07 (0.26) | 0.06 (0.23) | 0.07 (0.25) |
| Chronic pulmonary disease | 0.11 (0.31) | 0.28 (0.45) | 0.24 (0.43) | 0.28 (0.45) |
| Diabetes, uncomplicated | 0.14 (0.35) | 0.28 (0.45) | 0.24 (0.43) | 0.27 (0.45) |
| Diabetes, complicated | 0.04 (0.20) | 0.11 (0.32) | 0.09 (0.29) | 0.11 (0.31) |
| Hypothyroidism | 0.08 (0.27) | 0.43 (0.49) | 0.44 (0.50) | 0.44 (0.50) |
| Renal failure | 0.02 (0.15) | 0.09 (0.28) | 0.07 (0.25) | 0.09 (0.28) |
| Liver disease | 0.01 (0.12) | 0.04 (0.21) | 0.04 (0.20) | 0.04 (0.21) |
| Peptic ulcer disease excluding bleeding | 0.01 (0.11) | 0.04 (0.19) | 0.03 (0.17) | 0.04 (0.19) |
| AIDS/H1V | <0.01 (0.02) | <0.01 (0.03) | <0.01 (0.02) | <0.01 (0.03) |
| Lymphoma | <0.01 (0.07) | 0.01 (0.11) | 0.01 (0.10) | 0.01 (0.11) |
| Metastatic cancer | 0.01 (0.09) | 0.03 (0.16) | 0.02 (0.14) | 0.03 (0.16) |
| Solid tumor without metastasis | 0.07 (0.26) | 0.17 (0.38) | 0.15 (0.36) | 0.17 (0.38) |
| Rheumatoid arthritis/ collagen vascular diseases | 0.03 (0.17) | 0.10 (0.30) | 0.09 (0.29) | 0.10 (0.30) |
| Coagulopathy | 0.01 (0.12) | 0.06 (0.24) | 0.05 (0.22) | 0.06 (0.24) |
| Obesity | 0.03 (0.16) | 0.06 (0.23) | 0.05 (0.22) | 0.06 (0.23) |
| Weight loss | 0.01 (0.12) | 0.08 (0.28) | 0.10 (0.30) | 0.09 (0.28) |
| Fluid and electrolyte disorders | 0.04 (0.21) | 0.18 (0.39) | 0.16 (0.36) | 0.18 (0.38) |
| Blood loss anemia | 0.01 (0.07) | 0.03 (0.16) | 0.02 (0.15) | 0.03 (0.16) |
| Deficiency anemia | 0.03 (0.16) | 0.12 (0.33) | 0.10 (0.31) | 0.12 (0.32) |
| Alcohol abuse | 0.01 (0.07) | 0.01 (0.09) | 0.01 (0.08) | 0.01 (0.09) |
| Drug abuse | <0.01 (0.04) | 0.01 (0.07) | <0.01 (0.07) | 0.01 (0.07) |
| Psychoses | 0.01 (0.10) | 0.02 (0.16) | 0.02 (0.13) | 0.02 (0.15) |
| Depression | 0.05 (0.22) | 0.15 (0.36) | 0.12 (0.33) | 0.15 (0.35) |
|  |  |  |  |  |
| N | 3,403,919 | 47,412 | 19,852 | 23,846 |
| N Graves Disease | 23,846 | 23,706 | 19,852 | 23,846 |
| N Alzheimer's Disease | 321,244 | 5,121 | 2,120 | 2,856 |
| N Dead | 1,416,903 | 22,556 | 7,912 | 10,841 |
|  | | | | |
| Note: Numbers presented are sample means with standard deviations in parentheses. | | |  |  |

| **Supplementary Table 2. Propensity Score Matching Quality and Group Comparison for Unconfirmed Graves**    **Unmatched**  **Matched**  **Unconfirmed** | | | |
| --- | --- | --- | --- |
|  | **Full Sample** | **Full Sample** | **Graves*** |
| Baseline Age | **63.94** | -6.66 | **-22.15** |
| Male | **-37.28** | -2.80 | -7.07 |
| White | -4.44 | 6.92 | -0.97 |
| Black | 6.35 | -3.98 | 1.91 |
| Hispanic | -0.60 | -4.07 | -3.08 |
| Other | 2.03 | -1.69 | 0.23 |
| Ever Dual Eligible | 7.07 | **-11.70** | -5.64 |
| Yearly Trend (2000=0) | **-34.23** | **-14.54** | **-11.60** |
| Congestive heart failure | **44.16** | -4.71 | -7.90 |
| Cardiac arrhythmias | **57.14** | -3.90 | -1.86 |
| Valvular disease | **44.02** | -1.50 | -8.66 |
| Pulmonary circulation Disorders | **21.33** | -0.90 | -1.39 |
| Peripheral vascular disorders | **42.47** | -3.21 | **-11.12** |
| Hypertension, uncomplicated | **66.35** | 2.05 | **-11.64** |
| Hypertension, complicated | **40.80** | -2.45 | -8.61 |
| Paralysis | **10.97** | -2.32 | -2.97 |
| Other neurological disorders | **21.34** | -4.31 | -4.18 |
| Chronic pulmonary disease | **43.40** | -3.32 | -8.25 |
| Diabetes, uncomplicated | **33.39** | -4.52 | -8.10 |
| Diabetes, complicated | **26.38** | -4.20 | -5.44 |
| Hypothyroidism | **92.70** | 5.18 | -1.72 |
| Renal failure | **28.77** | -2.07 | -6.30 |
| Liver disease | **17.69** | -1.39 | -0.95 |
| Peptic ulcer disease excluding bleeding | **17.91** | -1.09 | -4.56 |
| AIDS/H1V | 1.11 | -1.29 | -1.16 |
| Lymphoma | 8.59 | -0.67 | -2.06 |
| Metastatic cancer | **14.26** | -2.84 | -4.04 |
| Solid tumor without metastasis | **31.83** | -1.93 | -6.34 |
| Rheumatoid arthritis/ collagen vascular diseases | **29.14** | -2.53 | -3.76 |
| Coagulopathy | **24.97** | -1.93 | -3.99 |
| Obesity | **15.66** | -1.27 | -1.36 |
| Weight loss | **33.29** | -1.44 | 5.26 |
| Fluid and electrolyte disorders | **44.26** | -3.45 | -6.26 |
| Blood loss anemia | **16.98** | 0.08 | -1.38 |
| Deficiency anemia | **36.01** | -2.83 | -4.90 |
| Alcohol abuse | 2.87 | -2.15 | -1.04 |
| Drug abuse | 5.72 | -0.70 | -0.33 |
| Psychoses | **11.46** | -2.41 | -4.16 |
| Depression | **32.66** | -2.87 | -6.52 |
|  |  |  |  |
| N | 3,403,919 | 47,412 | N.A. |
| N Graves Disease | 23,846 | 23,706 |  |
| N Alzheimer's Disease | 321,244 | 5,121 |  |
|  | | | |
| Note: Numbers presented are standardized differences; Standardized differences with an absolute value greater than 10 are in bold. | | | |
| * Standardized differences in this column compare the confirmed/unconfirmed Graves disease groups. Sign indicates whether the mean for the unconfirmed group is greater/less than the mean of the confirmed group. | | | |

| **Supplementary Table 3. Logistic Group Membership Models**    **Graves Disease Algorithm Type** | | | | | | |
| --- | --- | --- | --- | --- | --- | --- |
|  | **Confirmed** | | | **Unconfirmed** | | |
|  | **Estimate** | **Standard Error** | **p-val** | **Estimate** | **Standard Error** | **p-val** |
| Intercept | -9.8233 | 0.0771 | <.0001 | -11.4756 | 0.0676 | <.0001 |
| Baseline age | 0.0515 | 0.0010 | <.0001 | 0.0727 | 0.0009 | <.0001 |
| Male | -0.5138 | 0.0184 | <.0001 | -0.2318 | 0.0164 | <.0001 |
| Black | 0.4783 | 0.0252 | <.0001 | 0.3873 | 0.0238 | <.0001 |
| Hispanic | -0.0608 | 0.0681 | 0.3722 | 0.1254 | 0.0560 | 0.0252 |
| Other | 0.4026 | 0.0343 | <.0001 | 0.4295 | 0.0318 | <.0001 |
| Ever Dual Eligible | -0.2104 | 0.0199 | <.0001 | -0.1660 | 0.0177 | <.0001 |
| Yearly Trend (2000=0) | -0.0421 | 0.0010 | <.0001 | -0.0325 | 0.0009 | <.0001 |
| Congestive heart failure | 0.0225 | 0.0237 | 0.3434 | -0.0271 | 0.0207 | 0.1905 |
| Cardiac arrhythmias | 0.7126 | 0.0185 | <.0001 | 0.5074 | 0.0169 | <.0001 |
| Valvular disease | 0.3311 | 0.0238 | <.0001 | 0.5122 | 0.0205 | <.0001 |
| Pulmonary circulation Disorders | 0.0929 | 0.0411 | 0.0236 | 0.0027 | 0.0372 | 0.9428 |
| Peripheral vascular disorders | 0.1774 | 0.0227 | <.0001 | 0.3084 | 0.0192 | <.0001 |
| Hypertension, uncomplicated | 0.2759 | 0.0168 | <.0001 | 0.4749 | 0.0159 | <.0001 |
| Hypertension, complicated | 0.3882 | 0.0258 | <.0001 | 0.4830 | 0.0221 | <.0001 |
| Paralysis | 0.0213 | 0.0591 | 0.7186 | 0.0748 | 0.0494 | 0.1299 |
| Other neurological disorders | 0.0456 | 0.0340 | 0.1797 | 0.0892 | 0.0295 | 0.0025 |
| Chronic pulmonary disease | 0.2719 | 0.0187 | <.0001 | 0.3809 | 0.0166 | <.0001 |
| Diabetes, uncomplicated | 0.0740 | 0.0208 | 0.0004 | 0.2009 | 0.0184 | <.0001 |
| Diabetes, complicated | 0.0333 | 0.0309 | 0.2813 | 0.0357 | 0.0268 | 0.1828 |
| Hypothyroidism | 1.6577 | 0.0161 | <.0001 | 1.6816 | 0.0148 | <.0001 |
| Renal failure | -0.3735 | 0.0345 | <.0001 | -0.2790 | 0.0293 | <.0001 |
| Liver disease | 0.1835 | 0.0387 | <.0001 | 0.1936 | 0.0352 | <.0001 |
| Peptic ulcer disease excluding bleeding | 0.4161 | 0.0443 | <.0001 | 0.5607 | 0.0369 | <.0001 |
| AIDS/H1V | -0.4956 | 0.3661 | 0.1758 | 0.0338 | 0.2599 | 0.8965 |
| Lymphoma | 0.0230 | 0.0733 | 0.7536 | 0.1610 | 0.0624 | 0.0098 |
| Metastatic cancer | 0.0435 | 0.0560 | 0.4374 | 0.2428 | 0.0461 | <.0001 |
| Solid tumor without metastasis | 0.4696 | 0.0221 | <.0001 | 0.5759 | 0.0194 | <.0001 |
| Rheumatoid arthritis/ collagen vascular diseases | 0.3689 | 0.0265 | <.0001 | 0.4649 | 0.0235 | <.0001 |
| Coagulopathy | 0.2493 | 0.0358 | <.0001 | 0.3197 | 0.0311 | <.0001 |
| Obesity | -0.2400 | 0.0344 | <.0001 | -0.1831 | 0.0312 | <.0001 |
| Weight loss | 1.1233 | 0.0271 | <.0001 | 0.7361 | 0.0270 | <.0001 |
| Fluid and electrolyte disorders | 0.0937 | 0.0237 | <.0001 | 0.1102 | 0.0208 | <.0001 |
| Blood loss anemia | 0.2201 | 0.0522 | <.0001 | 0.1479 | 0.0468 | 0.0016 |
| Deficiency anemia | 0.2244 | 0.0268 | <.0001 | 0.2529 | 0.0235 | <.0001 |
| Alcohol abuse | -0.3713 | 0.0919 | <.0001 | -0.2839 | 0.0797 | 0.0004 |
| Drug abuse | -0.0638 | 0.1085 | 0.5563 | -0.1302 | 0.0983 | 0.1854 |
| Psychoses | -0.2146 | 0.0581 | 0.0002 | -0.1171 | 0.0475 | 0.0136 |
| Depression | 0.0124 | 0.0241 | 0.6078 | 0.1802 | 0.0211 | <.0001 |
|  | | | | | | |
|  | | | | | | |


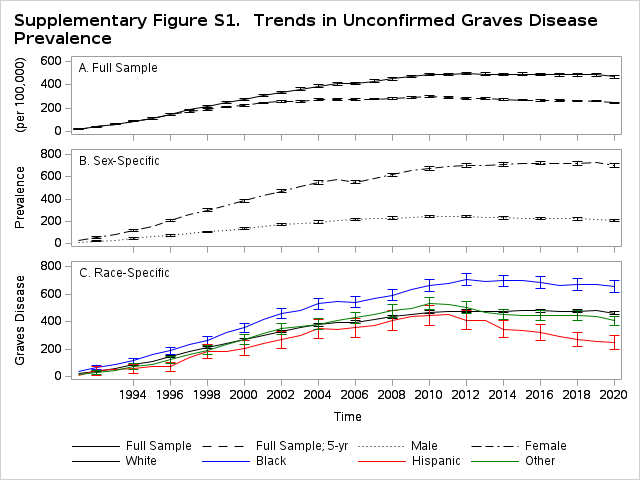


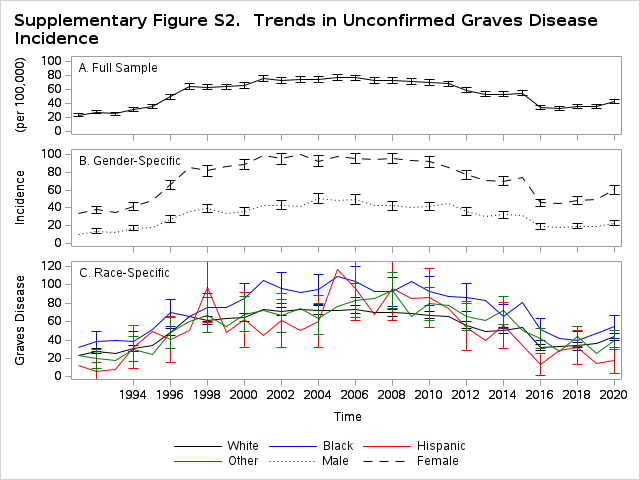

Supplement: Supplementary file 1 — Additional file 1: Supplementary Appendix A. Supplementary Table 1. Summary Statistics for Unconfirmed Graves Disease. Supplementary Table 2. Propensity Score Matching Quality and Group Comparison for Unconfirmed Graves. Supplementary Table 3. Logistic Group Membership Models. Supplementary Figure S1. Trends in Unconfirmed graves disease prevalence. Supplementary Figure S2. Trends in Unconfirmed graves disease incidence. [file 40842_2024_170_MOESM1_ESM.docx]
